# Gene expression profiles among murine strains segregate with distinct differences in the progression of radiation-induced lung disease

**DOI:** 10.1242/dmm.028217

**Published:** 2017-04-01

**Authors:** Isabel L. Jackson, Fitsum Baye, Chirayu P. Goswami, Barry P. Katz, Andrew Zodda, Radmila Pavlovic, Ganga Gurung, Don Winans, Zeljko Vujaskovic

**Affiliations:** 1 Division of Translational Radiation Sciences, Department of Radiation Oncology, University of Maryland School of Medicine, Baltimore, MD 21202, USA; 2 Department of Biostatistics, Indiana University School of Medicine and Richard M. Fairbanks School of Public Health, Indianapolis, IN 46202, USA; 3 Thomas Jefferson University Hospital, Molecular and Genomic Pathology Lab, Philadelphia, PA 19107, USA

**Keywords:** Radiation pneumonitis, Lung fibrosis, Gene expression profiling, Murine strain differences

## Abstract

Molecular mechanisms underlying development of acute pneumonitis and/or late fibrosis following thoracic irradiation remain poorly understood. Here, we hypothesize that heterogeneity in disease progression and phenotypic expression of radiation-induced lung disease (RILD) across murine strains presents an opportunity to better elucidate mechanisms driving tissue response toward pneumonitis and/or fibrosis. Distinct differences in disease progression were observed in age- and sex-matched CBA/J, C57L/J and C57BL/6J mice over 1 year after graded doses of whole-thorax lung irradiation (WTLI). Separately, comparison of gene expression profiles in lung tissue 24 h post-exposure demonstrated >5000 genes to be differentially expressed (*P*<0.01; >twofold change) between strains with early versus late onset of disease. An immediate divergence in early tissue response between radiation-sensitive and -resistant strains was observed. In pneumonitis-prone C57L/J mice, differentially expressed genes were enriched in proinflammatory pathways, whereas in fibrosis-prone C57BL/6J mice, genes were enriched in pathways involved in purine and pyrimidine synthesis, DNA replication and cell division. At 24 h post-WTLI, different patterns of cellular damage were observed at the ultrastructural level among strains but microscopic damage was not yet evident under light microscopy. These data point toward a fundamental difference in patterns of early pulmonary tissue response to WTLI, consistent with the macroscopic expression of injury manifesting weeks to months after exposure. Understanding the mechanisms underlying development of RILD might lead to more rational selection of therapeutic interventions to mitigate healthy tissue damage.

## Abstract (precis)

**Summary:** Rational mouse model selection is crucial for identifying new therapeutic targets and screening medical interventions in acute pneumonitis and/or late fibrosis following thoracic irradiation.

## INTRODUCTION

Radiation-induced lung disease (RILD) remains the most common healthy tissue complication associated with radiation treatment of thoracic tumors. The disease is defined by two distinct phases, pneumonitis and fibrosis, that are separated in both time and histopathological sequelae (Travis, 1987). Radiation pneumonitis affects 5-15% of patients undergoing thoracic radiotherapy. It is defined as an early, transient phase that occurs between 1 and 7 months after exposure, with a peak incidence at 3-4 months. Development of pneumonitis during the course of treatment or shortly thereafter can potentially compromise cancer cure and, in rare instances, be life-threatening (Williams et al., 2010). In contrast, pulmonary fibrosis is more common and affects ≥50% patients treated with radiation for thoracic tumors, including lung cancers, breast cancers and mediastinal lymphomas (Appelt et al., 2014). Fibrosis is progressive, and clinical manifestations occur months to years after completion of therapy, with symptoms ranging from nonproductive cough to dyspnea on exertion.

Despite decades of research, no U.S. Food and Drug Administration-approved therapies are available to prevent, mitigate, and/or treat radiation pneumonitis and/or fibrosis; nor do well-defined biological markers predict individual risk for development of disease. This is, in part, a result of the biological complexity of RILD, in which injurious mechanisms begin at the time of exposure and progress through a clinically latent period before overt onset of pneumonitis and/or fibrosis (Bentzen, 2006). Progressive fibrosis has been observed to occur in the absence of clinically symptomatic radiation pneumonitis. In experimental models, the ability to dissociate radiation pneumonitis from fibrosis by dose fractionation and pharmaceutical interventions suggests that these two pathologies might be distinct and result from independent (although perhaps overlapping) underlying mechanisms of injury (Travis and Tucker, 1986).

It is well known that murine models of RILD display broad heterogeneity in temporal onset, radiation dose response, and phenotypic expression of disease, reflecting variations observed in humans. Over the past decade, the majority of preclinical studies have used a survival endpoint of 120-180 days for evaluation of therapeutic interventions against RILD. However, because of the protracted latency period between time of exposure and development of RILD in some strains, the use of survival endpoints ≤180 days might not permit full progression of disease, leading to bias in data interpretation. Further, few studies take into consideration animal age at time of irradiation or sex-based differences in pulmonary radiation response, each of which can confer strong variation in severity and incidence of pneumonitis and fibrosis following thoracic radiation exposure.

We previously reported on the dose­-response relationship and pathophysiological comparability of RILD in three murine strains (CBA/J, C57BL/6J and C57L/J), nonhuman primates (NHPs), and humans over the first 180 days after exposure (Jackson et al., 2014). Our study design and strain selection were informed by three decades of preclinical research in RILD (Sharplin and Franko, 1989a,b; Terry et al., 1988; Travis et al., 1981, 1980). Consistent with earlier reports, the predominant histological feature in moribund C57L/J and CBA/J mice was an acute pneumonitis over dose ranges of 9.0-13.0 and 13-16 Gy, respectively. In the C57BL/6J strain, the lungs displayed scarred, retracted fibrosis over a dose range of 12.5-15 Gy (Jackson et al., 2010, 2011, 2012, 2014).

In this study we expand on our previous findings to report on the natural history of disease progression up to 1 year after thoracic irradiation and define the genes and/or pathways that segregate to ‘pneumonitis-prone’ versus ‘fibrosis-prone’ mice using differential gene expression analysis. The data demonstrate significant differences in dose response, time to disease onset, and phenotype of injury. Moreover, ultrastructural damage and gene expression profiles suggest that tissue response to radiation within the first 24 h determines tissue fate. Taken together, we report the importance of appropriate strain selection, control over biological variables, and sufficient follow-up time to accurately identifying new therapeutic targets and testing of new medical interventions.

## RESULTS

### Natural history of disease progression for RILD in CBA/J, C57L/J, and C57BL/6J mice

Longitudinal studies were performed to assess the progression of RILD over a 1-year (360-day) period post-exposure using signs of major morbidity and/or mortality as the primary endpoint. Secondary endpoints to assess signs and severity of lung damage included qualitative and quantitative indices of pulmonary function, edema and/or congestion, and histopathological damage.

**Fig. 1.**
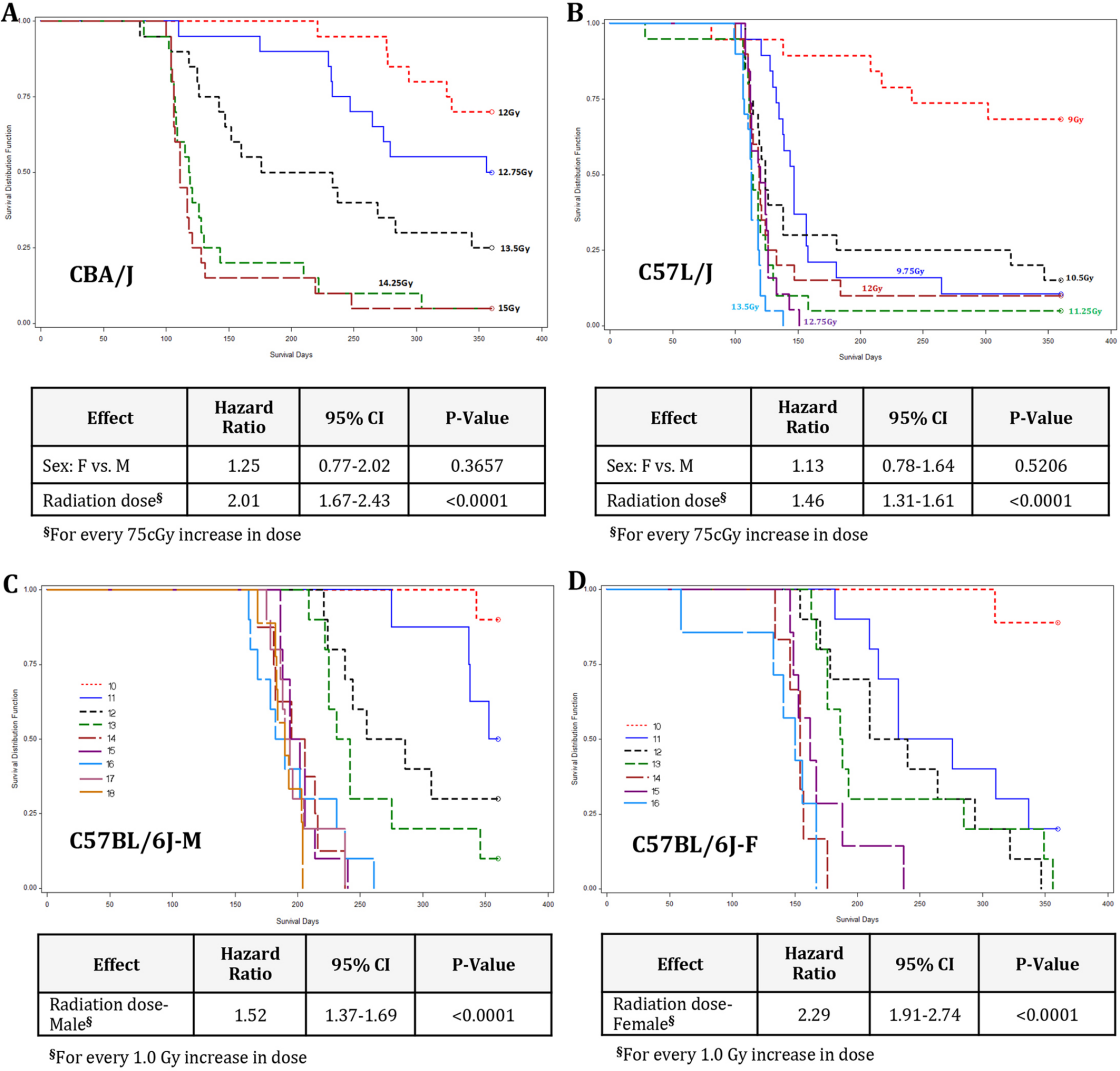
**Kaplan–Meier curves for survival to 360** **days** **following whole-thorax lung irradiation.** Survival curves for (A) age- and sex-matched CBA/J; (B) age- and sex-matched C57L/J; (C) male C57BL/6J; and (D) female C57BL/6J mice. Cox proportional hazard regression analysis was performed to determine the association between radiation dose and time to survival, and the radiation dose by sex interaction effect. Risk of dying (hazard ratio, HR) with 95% lower and upper confidence intervals (CI) are presented. Each radiation dose group was composed of 20 age-matched animals (50% male, 50% female).

Data demonstrate that in pneumonitis-prone CBA/J mice, animal sex had no significant effect on mortality (*P*=0.80) or time to death (*P*=0.37; Fig. 1A). For every 75 cGy increase in radiation dose, the odds of death by day 360 increased by 2.9 times (95% CI: 1.86-4.61; *P*<0.0001).

No significant association between sex and time to death (*P*=0.52) or mortality (*P*=0.095) was noted among C57L/J mice (Fig. 1B). In this strain, the odds of death increased by 1.46 times (95% CI: 1.31-1.61) for every 75 cGy increase in radiation dose (*P*<0.0001).

In contrast, a significant sex by radiation dose interaction effect (*P*<0.0001) was seen in C57BL/6J (BL6) mice; therefore, the effect of radiation dose on time to death was evaluated separately by sex (Fig. 1C,D). For every 1 Gy increase in radiation dose, the odds of dying by day 360 also increased, but female B6 mice had a higher rate of death than males with increasing radiation dose. In this study, female C57BL/6J mice irradiated at a dose of 17-18 Gy were excluded from final analysis because of loss of animals from excessive barbering and ulcerative dermatitis (common in this strain and exacerbated by radiation).

### Dose and quantification of exposure: influence of murine strain, sex and radiation dose on risk of dying following thoracic irradiation

**Fig. 2.**
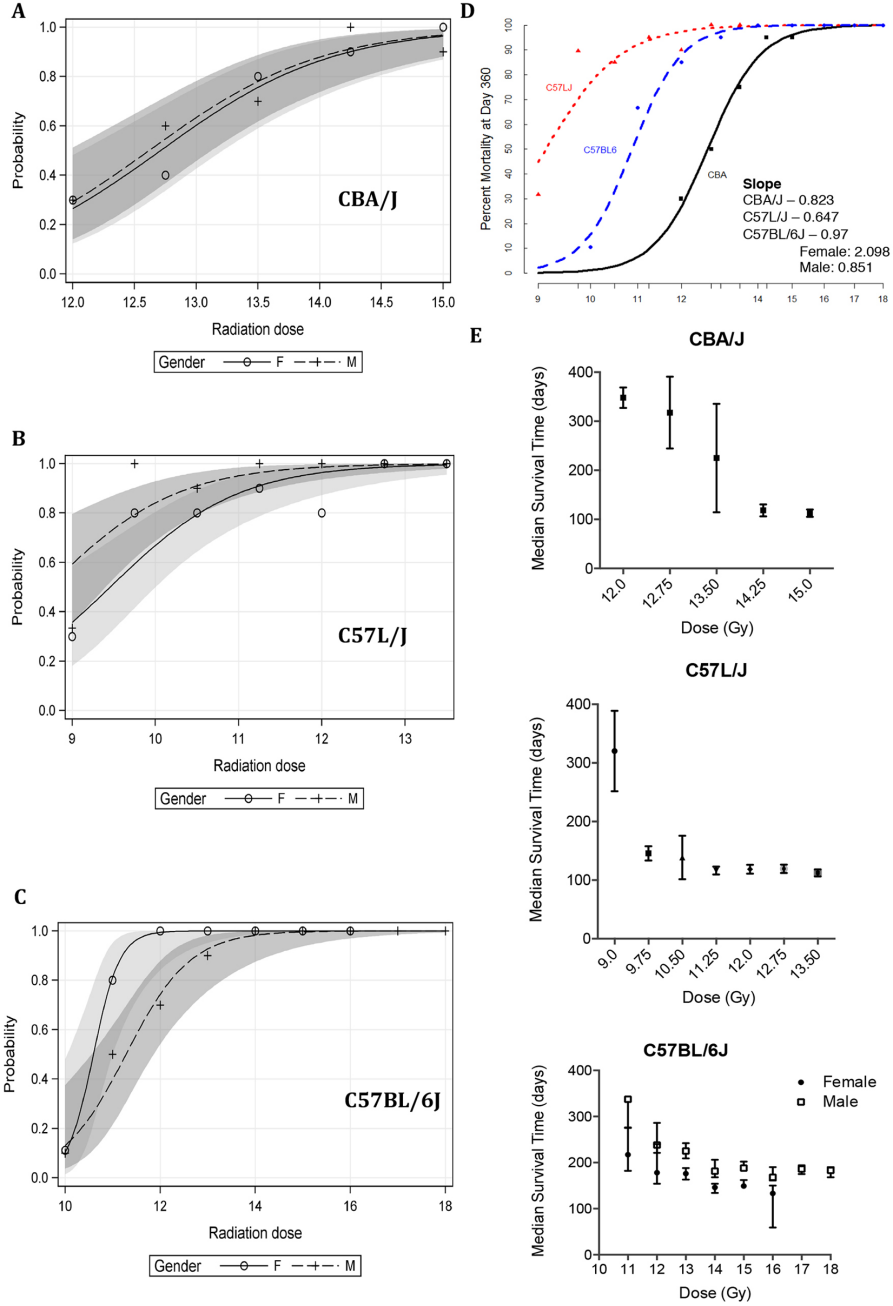
**Radiation dose response relationship and median survival times.** (A-C) Predicted probabilities for lethality to 360 days with 95% confidence limits for (A) CBA/J; (B) C57L/J; and (C) C57BL/6J mice by sex. (D) Dose-response relationship for survival to 360 days is right-shifted from C57L/J→CBA/J→C57BL/6J. (E) Median survival times for each strain. Data represent the median survival estimate± the upper and lower limit. Each radiation dose group comprised 20 age- and sex-matched animals. Only male C57BL/6J mice were included in the final analysis for 17-18 Gy radiation groups as all female mice within this dose range were excluded due to early euthanasia resulting from severe dermatitis prior to the onset of radiation pneumonitis/fibrosis.

Probit analysis was performed to determine the probability of major morbidity and/or mortality within the first 360 days post-exposure in each strain. There was a shift in the position of the dose-response curve across strains. The lethal dose for 50% of animals over the first 360 days (LD_50_/360) was 12.65 Gy (95% CI: 12.28-13.02) in sex-matched CBA/J mice (Fig. 2A) and 9.15 Gy (95% CI: 8.74-9.57) in sex-matched C57L/J mice, indicating greater pulmonary sensitivity in the latter strain (Fig. 2B). The LD_50_/360 for male C57BL/6J mice was 11.24 Gy (95% CI: 10.78-11.72) and for female C57BL/6J mice was 10.58 (95% CI: 10.28-10.89; Fig. 2C). Overlay of the dose-response curves for sex-matched mice is shown in Fig. 2D. In all strains, the rate of disease progression measured by median survival time was inversely related to radiation dose (Fig. 2E). At supralethal doses median survival time reached a plateau, after which an increase in radiation dose did not result in a shorter latency period.

### Clinical and pathological manifestations of radiation-induced lung injury (RILD) across strains

**Fig. 3.**
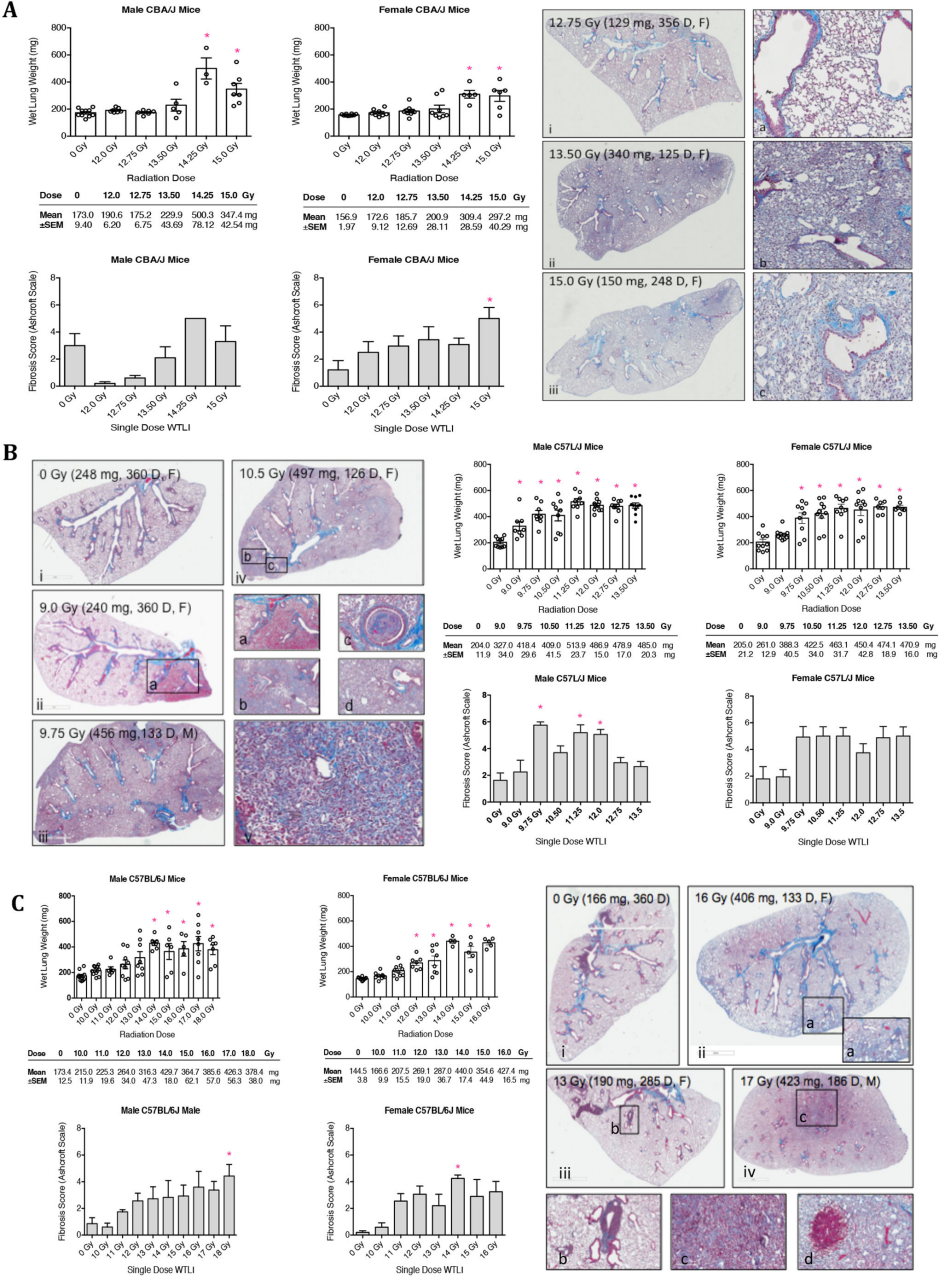
**Characterization of radiation-induced lung pathology.** (A) Wet lung weight, a marker of edema and congestion, is significantly increased in male and female CBA/J mice at 14.25 and 15 Gy whole-thorax lung irradiation (WTLI). Microscopic exam of Masson's trichrome-stained lung sections collected at time of major morbidity demonstrate significant inflammation and collagen deposition. (B) Significant increase is noted in wet lung weight in C57L/J mice starting at a WTLI dose of 9.0 Gy (male) and 9.75 Gy (female). The sensitivity of the lungs demonstrates a lack of a dose-response relationship for increasing lung inflammation, edema and congestion. Histology shows significant inflammation, airway congestion, and contracted fibrosis. Damage is significant across all dose levels. (C) In C57BL/6J mice, lung weight is increased at a dose of ≥14 Gy in male mice and ≥12.0 Gy in female mice, with a clear dose-response relationship for increased fibrosis. Hemorrhage was observed at the highest doses of 17 Gy. The dominant histological feature in this strain was subpleural fibrosis. Data represent mean±s.e.m. *n*=10 per sex in the 0 Gy cohort; *n*=5-10 per sex for each radiation dose cohort. Wet lung weight and histology from animals found dead were not included in the final analysis. Panels are labeled with the radiation dose (Gy), study day, lung weight (mg) at necropsy and animal sex (F/M). *P<0.05 by vs 0 Gy.

There was a dose-dependent increase in wet lung weight, consistent with edema and congestion, in all three strains. In CBA/J mice, histological features included increased alveolar wall thickness (Fig. 3Ai,ia), edema/congestion and macrophage accumulation (Fig. 3Aii,iib), and alveolar consolidation without contracted fibrosis (Fig. 3Aiii,iiic). Histological examination of lung tissue from C57L/J mice demonstrated greater cellular (Fig. 3iv,3iic), edema/congestion (Fig. 3Bii,iia-c), areas of involvement (Fig. 3Biii,iv), epithelial hyperplasia in bronchioles, and fibrosis (Fig. 3v) than either CBA/J or C57BL/6J mice. Furthermore, several animals displayed marked to severe accumulation of alveolar macrophages, consistent with acute pneumonitis and fibrosis.

Typical pathological findings in C57BL/6J were mild to moderate diffuse accumulation of alveolar macrophages with mild to moderate septal thickening and fibrosis (Fig. 3Cii). Less area of involvement was seen in the C57BL/6J strain than in the C57L/J strain (Fig. 3Cii,iii). In the C57BL/6J strain, there was little evidence of bronchiolar epithelial hyperplasia. In both C57L/J and C57BL/6J mice, a positive correlation was noted between alveolar macrophage accumulation and fibrosis (*P*<0.001). Pleural effusions (>0.5 g pleural fluid accumulation) in CBA/J mice were primarily observed over a narrow radiation dose range of 12.75-13.5 Gy. In male C57BL/6J mice, effusions were seen across all doses but were primarily confined to a dose range of 10-13 Gy. In female C57BL/6J mice, the majority of effusions occurred at doses of 11-12 Gy, where ∼70% of mice in each of those dose groups displayed effusions that likely contributed to mortality. Consistent with our previous studies, pleural effusions were not observed in C57L/J mice.

### Progression and pathobiology of RILD following whole-thorax lung irradiation (WTLI)

**Fig. 4.**
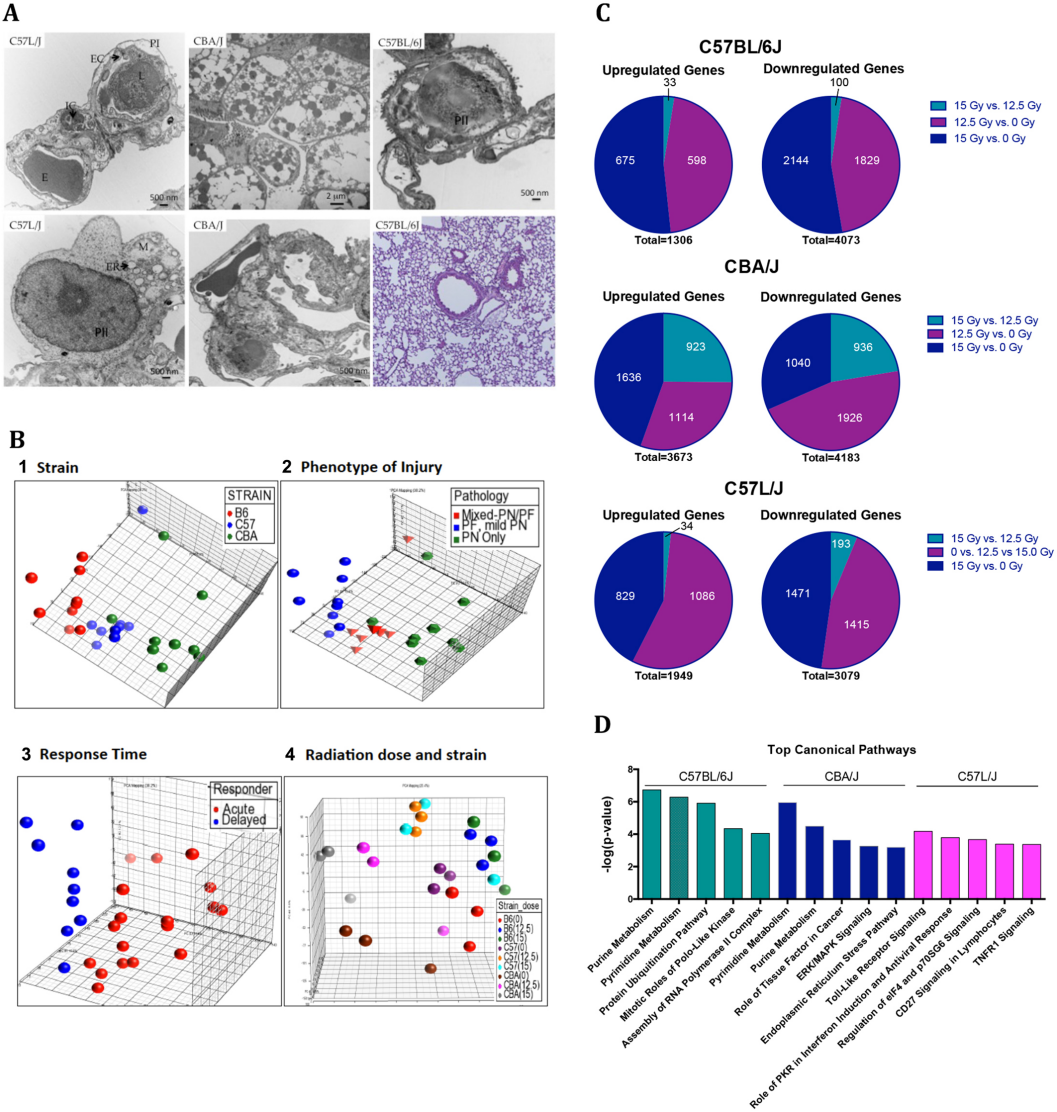
**Lung injury profile among strains at 24 h post-exposure to 0, 12.5, or 15** **Gy whole-lung thorax irradiation.** (A) Transmission electron micrographs demonstrate prominent ultrastructural differences among C57L/J, CBA/J and C57BL/6J mice 24 h after thoracic irradiation with a single dose of 15 Gy (*n*=3/strain). In C57L/J mice, prominent endothelial (EC) and epithelial cell (P1/PII) swelling with capillary occlusion is noted. Interstitial cell (IC) necrosis and inflammatory cell infiltrates are also seen. Abundant lethal cell injury and apoptosis, characterized by cytoplasmic blebbing, nuclear membrane invagination, mitochondrial (M), and endoplasmic reticulum (ER) swelling, are noted. Bronchial epithelial cells are severely swollen and apoptotic in the lungs of both C57L/J and CBA/J mice. The primary ultrastructural features in C57BL/6J lungs are significant interstitial edema and mild epithelial and endothelial cell swelling. Inflammation is not as prominently observed in C57BL/6J mice as in C57L/J. E, erythrocyte; L, lymphocyte. (B) Unsupervised principal component analysis (PCA) of lung tissue samples demonstrates clustering of genes according to (1) strain; (2) expected pathology at 12.5-15 Gy (i.e. pneumonitis only, mixed pneumonitis and fibrosis, or fibrosis only); (3) median time of survival at 12.5-15 Gy (<140 or >140 days); and (4) radiation dose and strain. For PCA, C57L/J and CBA/J mice are defined as acute responders (average mortality <140 days) and C57BL/6J as delayed responders (average mortality >140 days). Grouping of individual tissue samples between the two groups suggests distinct differences in gene expression between acute and delayed responders. Close clustering of individual samples denotes the biological reproducibility of results. (C) Total number of upregulated and downregulated genes (*P*<0.01, >twofold change) between 0, 12.5 and 15 Gy in each strain suggests that a highly complex biological response is induced by radiation in lung tissue. (D) Top five IPA canonical pathways with the highest gene enrichment in each strain.

In a separate experiment, we examined ultrastructural damage in lung tissue at 24 h post-exposure to a single dose of 15 Gy WTLI to compare early tissue response to radiation across strains. At this time point, ultrastructural abnormalities were observed in all three strains, although histopathological changes were not yet evident under a light microscope (Fig. 4A). In C57L/J mice, findings were consistent with acute lung injury, including interstitial cell necrosis, lethal cell injury and apoptosis, epithelial denudation, and disruption of the basement membrane, all of which are indicative of injury that is unresolvable without therapeutic intervention. The major ultrastructural pathology in evaluated lungs of CBA/J mice was severe bronchial epithelial cell damage. In contrast, injury in C57BL/6J mice was less severe and primarily characterized by mild endothelial and epithelial cell swelling and interstitial edema. Bronchial epithelial damage was not observed in evaluated sections from C57BL/6J mice.

### Pathophysiological mechanisms of RILD elucidated through differential gene expression analysis across murine strains

To better understand the unique gene expression patterns among murine strains before and after radiation, unsupervised hierarchical cluster analysis was performed. Cluster analysis was performed in a blinded fashion without *a priori* knowledge of the data. Principal component analysis (PCA) of the gene expression data demonstrated distinct clusters dependent on strain, pathology, time to disease onset and radiation dose response (Fig. 4B). For response time, CBA/J and C57L/J were categorized as ‘acute responders’ based on their shorter median survival time, consistent with acute onset of pneumonitis, and C57BL/6J mice were categorized as ‘delayed responders’ because of the prolonged latency period prior to onset of clinical symptoms following WTLI and fibrotic phenotype at the dose range evaluated. We identified 5088 genes differentially expressed between acute and delayed responders (*P*<0.01; >twofold change in expression). Of these, 1445 genes were upregulated and 3642 were downregulated in acute responders in contrast to delayed responders. A total of 3781 genes were differentially expressed after 15 Gy single-dose irradiation to the thorax between C57BL/6J versus CBA and C57L/J mice (*P*<0.01; >twofold change). PCA showed co-clustering of gene expression in acute responders versus delayed responders.

Next, we compared differences in gene expression profiles between 0 and 12.5 Gy, 0 and 15 Gy, and 12.5 and 15 Gy in each of the three strains (Fig. 4C). To derive biological meaning from the given data sets, differentially expressed genes were analyzed for enrichment of functional annotation using Ingenuity Pathways Knowledge Base (QIAGEN, Redwood City, CA). The significance of association between genes from the dataset and the functional pathway was calculated by ingenuity pathway analysis (IPA) using a right-tailed Fisher exact test. Fig. 4D shows the top five highly enriched canonical pathways in each strain.

### Top toxicology pathways enriched in pneumonitis- and fibrosis-prone mice

**Fig. 5.**
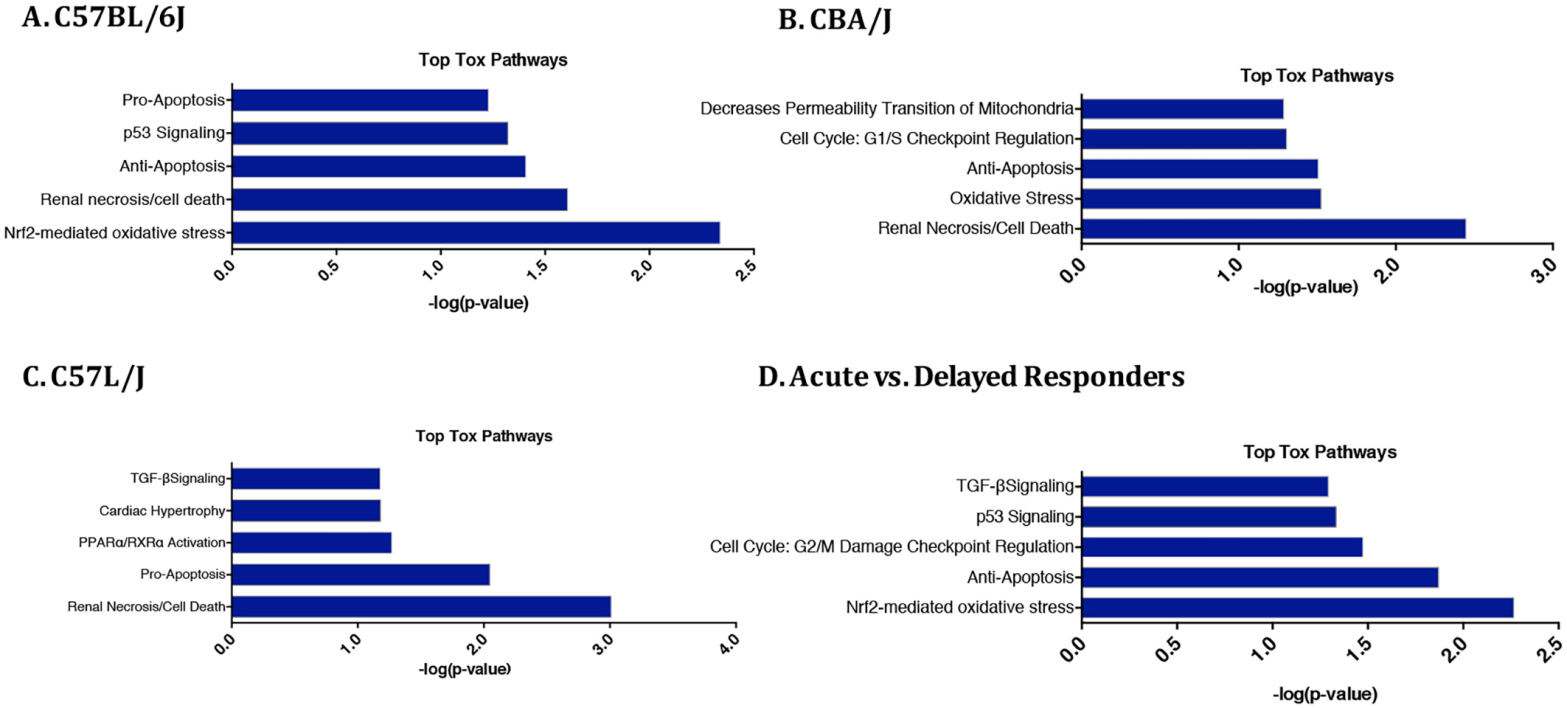
**Ingenuity Pathway Analysis of top toxic pathways associated with observed changes in gene expression.** Top five IPA toxicity pathways with the highest gene enrichment in (A) C57BL/6J, (B) CBA/J and (C) C57L/J mice, and (D) acute versus delayed responders. Renal necrosis/cell death is highly enriched in all three strains. In the relatively radiation-sensitive C57L/J strain, the pro-apoptosis pathway is enriched compared with the anti-apoptosis pathway in both CBA/J and C57BL/6J samples.

Fig. 5 shows the top five toxicology pathways with the highest gene enrichment as determined by IPA software in C57BL/6J, CBA/J and C57L/J strains and between acute and delayed responders. Top pathways significantly enriched in the acute and delayed responders data set, such as TGF-β signaling and Nrf2-mediated oxidative stress, have been previously reported to participate in radiation pathogenesis (Anscher et al., 2006, 2008; Mont et al., 2016; Travis et al., 2011).

### Gene expression profiles altered in pneumonitis- and non-pneumonitis-prone mice 24 h after radiation

In this study we identified the top differentially expressed genes between acute and delayed responders using an analysis of variance (ANOVA)-based approach. First, we identified the top genes differentially expressed between groups using an established cutoff of *P*<0.01 and >20% change in relative expression. Lists were then imported into IPA, and the top differentially expressed genes identified. Next, a gene search was performed using the Gene Ontology database (www.geneontology.org) to identify the biological process, cellular component and molecular function (not shown) associated with each of the top 20 differentially expressed genes. Differences in gene expression were found between acute phase response (*Serpina1* and *Orm1/2*), cell migration (*Vegfc* and *Fez1*), and angiogenesis (*Ang* and *Vegfc*). Table 1 lists the top upregulated and downregulated genes, their biological process, cellular component and significance.

**Table 1.**
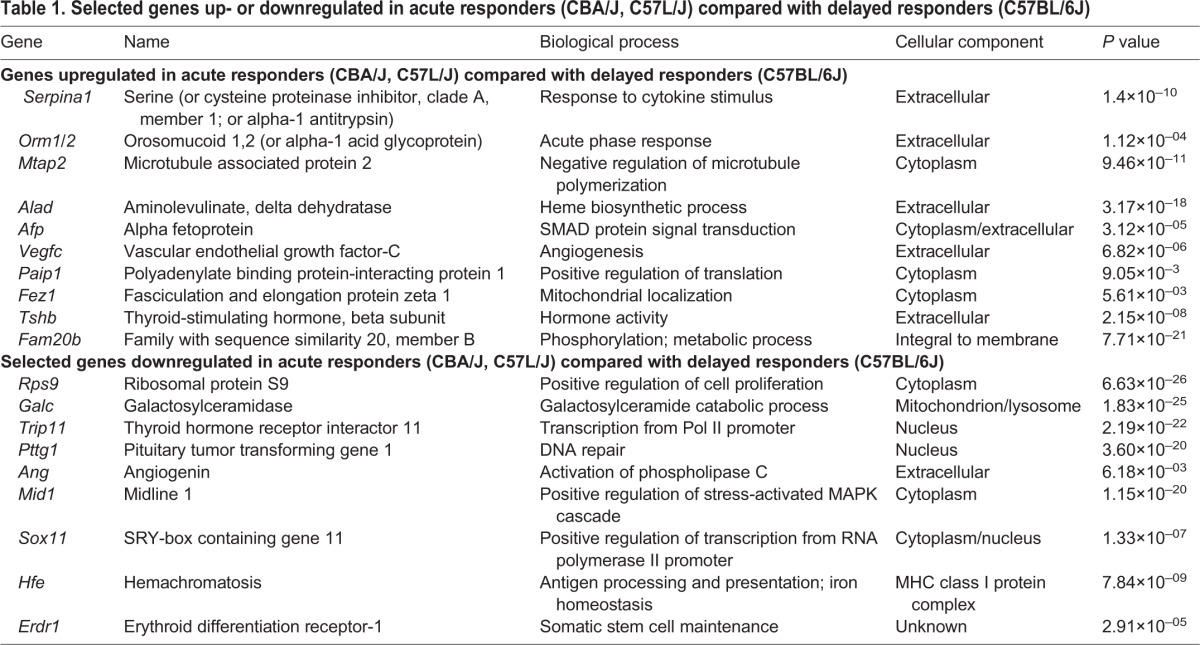
**Selected genes up- or downregulated in acute responders (CBA/J, C57L/J) compared with delayed responders (C57BL/6J)**

### Quantitative real-time PCR (qRT-PCR) of selected genes identified by IPA as differentially expressed among strains

qRT-PCR was performed using Assays on Demand (Thermo Fisher Scientific, Kansas City, MO) primers for 20 selected genes identified as differentially expressed between acute and delayed responders. qRT-PCR was performed on the same RNA previously used for microarray analysis. Data were normalized to the average of the 0 Gy control sample for each strain. Each biological sample was run independently in quadruplicate replicates. GAPDH was used as the reference gene.

**Fig. 6.**
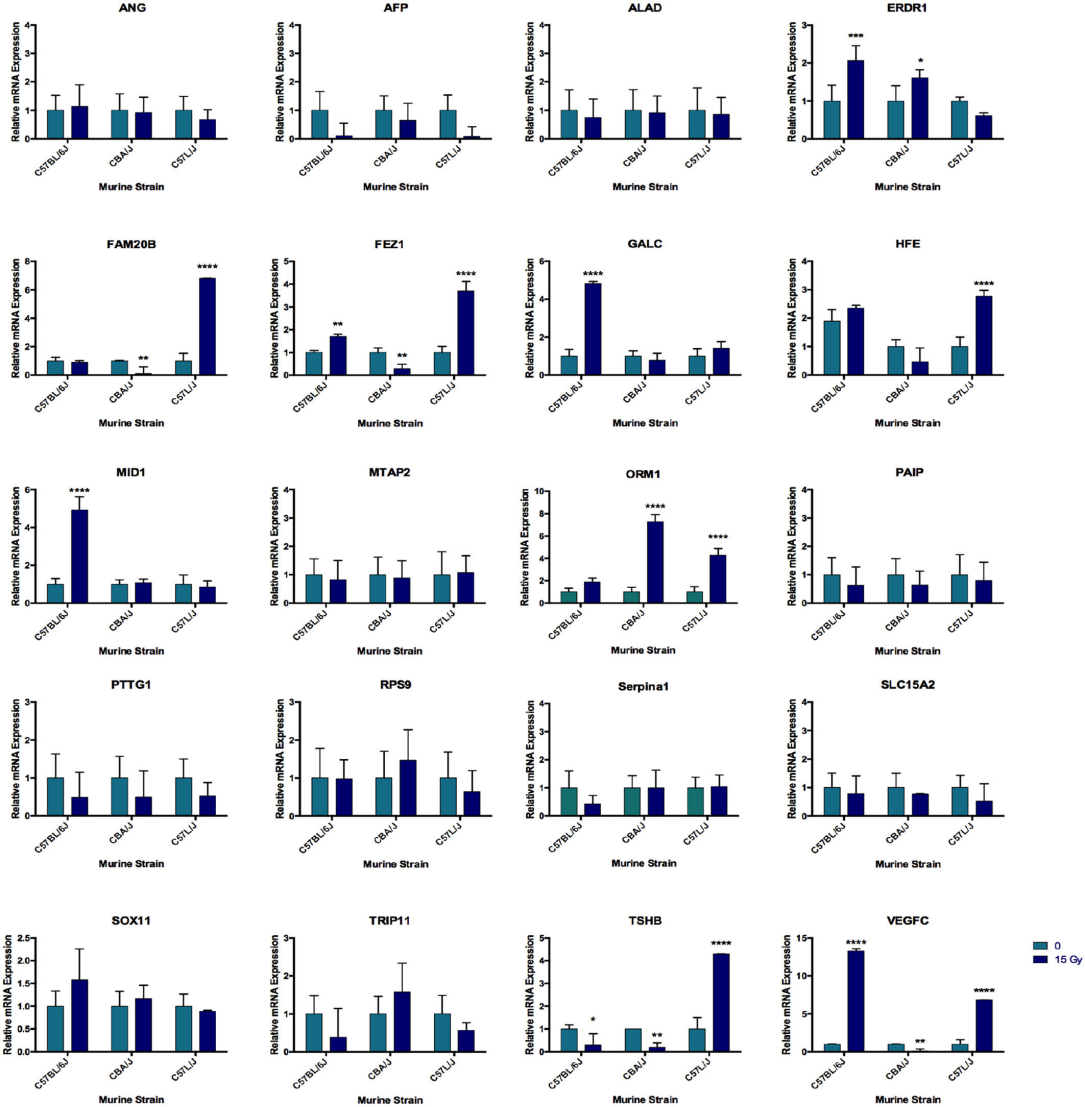
**Quantitative mRNA expression of selected genes identified by differential gene expression analysis.** Gene expression among the 20 selected genes identified by Ingenuity Pathway Analysis as being differentially expressed between acute (C57L/J, CBA/J) versus delayed (C57BL/6J) responders. qRT-PCR was performed using mRNA from the same tissue samples collected for microarray analysis. Relative mRNA expression for the irradiated (15 Gy) sample from each strain (C57BL/6J, CBA/J, C57L/J) was normalized to the respective sham-irradiated control. *n*=3-5 per group; data represent mean±s.e.m.; *P<0.05 **P<0.01; ***P<0.001, ****P<0.0001 between 15 Gy and 0 Gy.

Fig. 6 shows the relative mRNA expression of selected genes in sham (0 Gy) and irradiated (15 Gy) lungs from each strain. Two-way ANOVA with multiple comparisons test was used to evaluate statistical differences.

### Differences in messenger RNA and protein expression of acute phase proteins in early tissue response to radiation

**Fig. 7.**
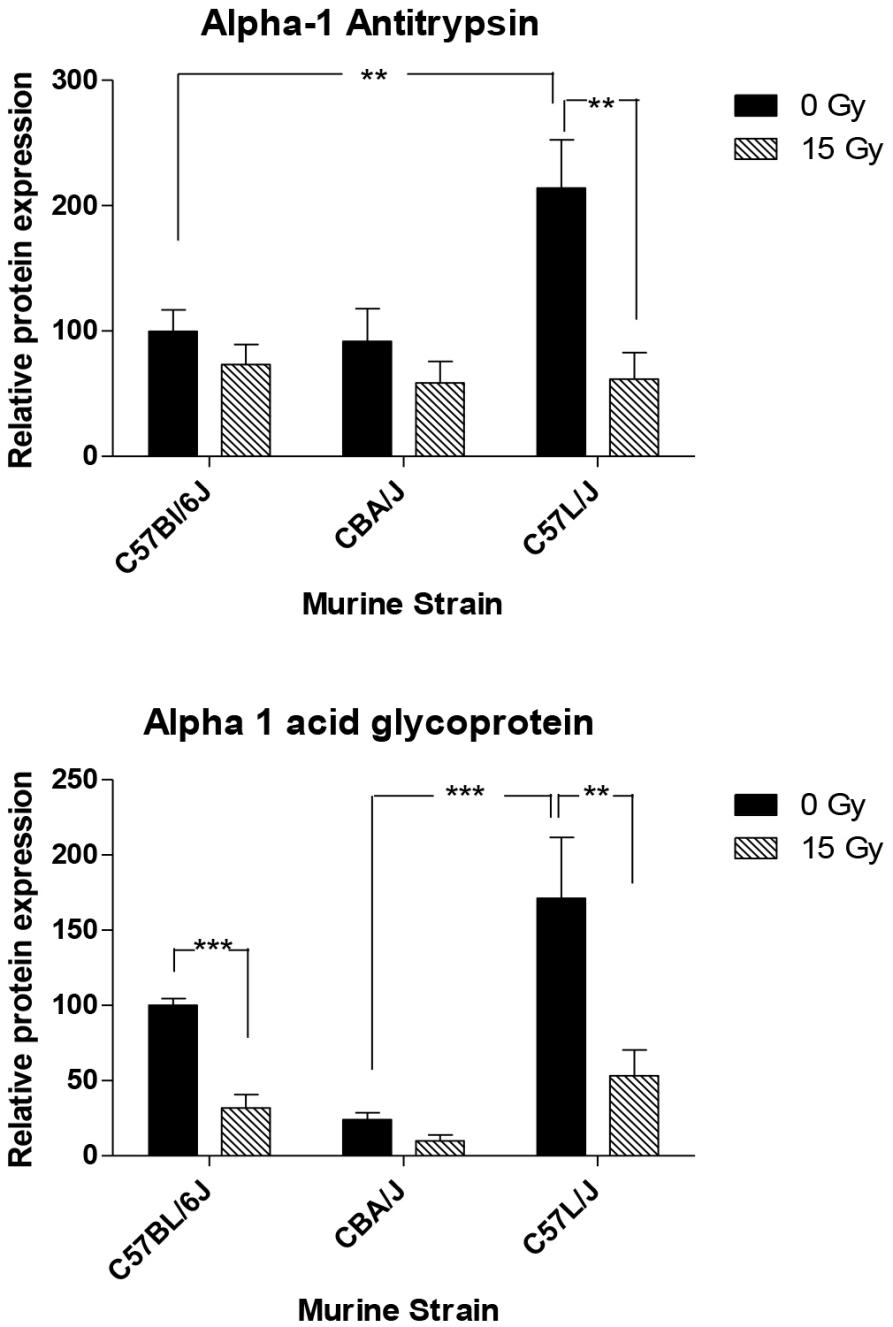
**Expression of acute phase proteins.** Expression of the protein product of *Serpina1*, alpha-1 antitrypsin (A1AT), and the protein product for *Orm1*, alpha-1-acid glycoprotein (AAG). *n*=5/group; Data presented as mean±s.e.m.; **P<0.01; ***P<0.001 by one-way ANOVA with multiple comparisons test.

Alpha-1 antitrypsin (A1AT) protein levels in the lungs of non-irradiated and irradiated mice were analyzed by western blot in a separate group of mice undergoing thoracic irradiation with a single dose of 15 Gy (*n*=5/group). Here, we found no difference in protein expression in the lungs of mice after irradiation, although there were higher basal levels in C57L/J when compared with C57BL/6J or CBA/J (Fig. 7A).

Western blot analysis was performed to determine changes in alpha-1 acid glycoprotein (AAG) expression (*n*=5/group). Higher basal levels of AAG were found in C57L than in CBA/J and C57BL/6J (Fig. 7B). Twenty-four hours after radiation, AAG was not increased; however, this might be due to the time needed for translation of protein from mRNA. A time-point study to evaluate AAG expression after irradiation might offer a clearer picture of alterations in AAG levels in the lungs of C57L/J, CBA/J and C57BL/6J mice. The higher basal levels of both A1AT and AAG in C57L/J mice might indicate that this strain is predisposed to inflammation.

## DISCUSSION

Elucidating the pathophysiological mechanisms that orchestrate the divergence of tissue response toward acute pneumonitis and/or fibrosis and identifying new therapeutic interventions requires well-designed, well-controlled preclinical studies with a stable and reproducible relationship between radiation dose and development of RILD. Preclinical study designs must take into consideration physical (e.g. radiation dose, geometry, volume), biological (e.g. species, strain, sex, age of animal models), and environmental (e.g. source colony or vendor, husbandry) parameters as these might significantly influence experimental outcomes (Conn, 2013; Justice and Dhillon, 2016).

In this study, we report on the natural history of disease progression in three murine models of WTLI and characterize differences in gene expression profiles associated with manifestation of radiation pneumonitis and/or fibrosis. One-year survival data indicate that lungs of C57L/J mice are strikingly more sensitive to radiation than either CBA/J or C57BL/6J mice over a dose range relevant to the threshold for RILD in humans (Fig. 1). Median survival times differed among strains with the C57BL/6J strain, displaying a protracted latency period compared with C57L/J mice, as previously described (Jackson et al., 2014).

Despite clinical recognition of age and sex differences in risk for radiation pneumonitis and fibrosis, these considerations are often overlooked in preclinical studies (Vogelius and Bentzen, 2012). Therefore, in this study, we compared the dose-response relationship in male and female mice in each strain. Data demonstrate that sex did not have a significant effect on pulmonary radiation response in either the C57L/J or CBA/J strain. However, in C57BL/6J mice there was a significant difference in time to disease progression between female and male mice (*P*<0.0001; Fig. 2). In this study, we controlled for animal age at the time of irradiation (10-12 weeks of age) and therefore did not assess the impact of age on pathogenesis of RILD. However, it is well known that individuals >54 years of age have an increased risk for developing pneumonitis and/or fibrosis following thoracic radiotherapy (Vogelius and Bentzen, 2012). This might result, in part, from compromise of pulmonary cardiac function caused by pre-existing comorbidities (e.g. chronic obstructive pulmonary disease) that were not modeled in this study.

Pathophysiological comparison of the models in this study suggests that transient changes in respiratory function consistent with the pneumonitis phase in the C57L/J strain (Jackson et al., 2010) are strikingly similar to those observed in human lungs (Marks et al., 2000). Furthermore, in humans, acute onset of injury with rapid progression to organ failure or recovery occurs 3-4 months after exposure. This is comparable with the time course of the pneumonitis phase observed in C57L/J mice in this study, where few deaths occurred 200-360 days post-exposure. Also similar is the observed lack of sex-dependent difference in dose response in this strain, as sex (male versus female; *P*=0.62) has not been observed as a risk factor for development of RILD in humans (Vogelius and Bentzen, 2012).

In this study, pleural effusions were observed in CBA/J and C57BL/6J but not C57L/J mice, primarily after 26 weeks post-WTLI. The relevance of effusions to lung injury in radiation cancer treatment is unclear, as pleural fluid accumulation is rarely seen in patients. However, Garofalo et al. found that NHPs develop significant pleural effusions following WTLI, which is mitigated by steroid (dexamethasone) treatment regimens (Garofalo et al., 2014). It is tempting to hypothesize that the use of dexamethasone as a standard of care for radiation pneumonitis in the clinic might explain why the pathology is not routinely observed.

Lung tissue collected at the time of scheduled or unscheduled euthanasia was examined microscopically for comparison of tissue damage among strains during early and late phases of injury. Acute radiation pneumonitis is characterized histologically by alveolar wall thickening, interstitial edema and congestion of the airways, inflammatory cell infiltration, epithelial denudation of the airways, and presence of hyaline membranes (Travis, 1987; Travis and Tucker, 1986). In this study, the lungs of CBA/J mice displayed inflammation characterized by macrophage accumulation and interstitial edema, along with moderate collagen deposition within the alveolar space at doses >13 Gy (Fig. 3A). Severe pneumonitis was observed in the lungs of moribund C57L/J mice, often with abundant fibrotic lesions (Fig. 3B). In the C57BL/6J strain, mild-to-moderate pneumonitis was observed at radiation doses ≥15 Gy; however, the predominant histological feature was localized-to-diffuse fibrosis, particularly around the large airways and subpleura (Fig. 3C).

Microscopic damage in irradiated lung tissue is rarely observed earlier than 6 weeks post-exposure. However, at 24 h after 15 Gy WTLI, clear differences in ultrastructural damage among strains were seen, suggesting an immediate difference in normal tissue sensitivity and tissue response to radiation among strains. In the relatively radiosensitive C57L/J strain, prominent swelling of endothelial and alveolar epithelial cells in the lung sections was observed, likely resulting in capillary occlusion that can affect blood flow to the tissue. Epithelial cell apoptosis and interstitial cell necrosis, along with lymphocyte infiltration, were also observed, suggestive of acute lung injury. In contrast, ultrastructural alterations in the lungs of C57BL/6J mice were mild with neither inflammation nor significant bronchiole epithelial damage observed in the examined tissue sections.

Gene expression profiling was performed to compare strain differences in pulmonary response to thoracic irradiation. Distinct strain-dependent differences consistent with heterogeneity in phenotypic expression of disease were observed (Fig. 4). Variation in gene enrichment to pathways such as Nrf2-mediated oxidative response and TGF-β1 signaling between and among strains suggests an immediate divergence in mechanisms underlying disease development and progression toward a pneumonitis and/or fibrosis phenotype (Fig. 5). Taken together, these data indicate the importance of selecting the appropriate murine model of WTLI for probing the mechanisms underlying RILD and testing new therapeutic interventions.

Here, gene expression profiling with microarrays identified the genes *Serpina1* (*P*<1.4×10^–10)^ and *Orm1* (*P*<1.12×10^–4^), which encode serine protease inhibitor A1AT and serine protease carrier AAG, respectively, as the top differentially expressed genes between acute (C57L/J, CBA/J) and late (C57BL/6J) responders (Fig. 6). Although differences in *Serpina1* expression were not statistically significant at *P*<0.05 using qRT-PCR, *Orm1* showed a statistically significant increase after radiation in both CBA/J (*P*<0.05) and C57L/J (*P*<0.001) mice. Protein expression was evaluated in lung tissue 24 h after sham irradiation or thoracic irradiation in C57BL/6J, CBA/J and C57L/J mice (*n*=5/strain and dose). The lack of correlation between mRNA and protein expression might result from the lag time between transcription and translation.

A literature search to compare pathophysiological findings and pathways of interest between our experimental model and human pulmonary response to radiation demonstrated that acute phase proteins have been previously implicated in radiation-induced normal tissue toxicity across species, including rodents, NHPs and humans. Zherbin et al. identified an increase in A1AT at the peak of radiation illness following total body irradiation in an NHP model (Zherbin et al., 1987). More recently, Jakobsson et al. observed an increase in both A1AT and AAG in the sera of patients with gastrointestinal toxicity following pelvic irradiation for anal or uterine cancer (Jakobsson et al., 2010). Using a bioinformatics approach, Oh et al. (2011) found a correlation between alpha-2 macroglobulin, also an acute-phase protein, and radiation pneumonitis in non-small cell lung cancer patients following fractionated radiation.

Our model of RILD differs from the clinical regimen in that wide-field, single doses of WTLI were delivered rather than localized, fractionated irradiation. However, prior studies have shown that phenotypic variation observed among murine strains extends to clinically relevant fractionation schemes and dose volumes. Nonetheless, WTLI is a useful model for establishing qualitative and quantitative endpoints to correlate pathophysiological mechanisms that orchestrate the divergence of tissue response with disease outcomes (e.g. pneumonitis and/or fibrosis).

In conclusion, data in this study point toward an immediate divergence in normal pulmonary tissue response to radiation among three murine strains with well-characterized differences in natural history of disease progression following thoracic irradiation.

## MATERIALS AND METHODS

### Animals

Experiments were conducted at Duke University (Durham, NC) and the University of Maryland School of Medicine (UMSOM, Baltimore, MD). All experiments were performed in compliance with the Animal Use Protocols approved by the Institutional Animal Care and Use Committee at each institution. To establish the natural history of disease progression across murine strains, age- and sex-matched C57L/J, CBA/J and C57BL/6J mice were purchased from Jackson Labs, Bar Harbor, ME, and allowed to acclimate for 2 weeks prior to radiation exposure. Age- and sex-matched sham-irradiated controls were included for comparison of normal lung tissue among mice. Animals were identified by ear tags with a unique ID number and cage card throughout the study. Animal holding rooms were maintained at 21±3°C with 30-70% relative humidity. A 12-h light/dark cycle was maintained with lights turned on at ∼07:00 h and off at ∼19:00 h. Animals were provided hyperchlorinated (10 ppm) water and fed 2018SX Teklad Global 18% Protein Extruded rodent diet *ad libitum* throughout the study.

### Whole-thorax lung irradiation (WTLI)

The X-RAD 320 irradiator (Precision X-ray Inc., North Branford, CT) was commissioned by a board-certified medical physicist following the guidance of Task Group 61 of the American Association of Physicists in Medicine (Ma et al., 2001). Quality assurance and quality control procedures were followed during each radiation run to ensure reproducibility of radiation output and accurate dose measurements.

Animals, 10-12 weeks of age, were allocated to groups of 20 (50% male, 50% female) to receive a single dose of uniform whole-lung exposure across the dose range to induce 0 to 100% lethality over the first 180 days post-exposure consistent with earlier studies (Jackson et al., 2014). Anesthetized animals (70-100 mg/kg ketamine, 10-20 mg/kg xylazine) were irradiated in the prone position with 320 kVp X-rays (HVL ∼1 mm Cu, filter=2.00 mm AI, dose rate=1.25±0.03 Gy/m) at UMSOM. Radiation was delivered to the thorax through adjustable apertures with 8 mm lead shielding of the head and abdomen. For sham irradiation, animals (20 per strain) were treated in the same way except that the radiation source was not activated.

### Respiratory function analysis

Respiratory function was assessed using the Buxco whole-body plethysmograph (Wilmington, NC) as previously described (Jackson et al., 2012). Lung function measurements were recorded on alternating weeks, beginning before the time of irradiation and continuing for up to 180 days post-exposure, and at the time of euthanasia (data not shown).

### Euthanasia criteria

Moribund mice were euthanized by sodium pentobarbital (>100 mg/kg) followed by bilateral thoracotomy after cessation of respiration for >1 min. Imminent morbidity was determined by ≥20% body mass loss (single criteria) or if the animal met at least three of the following criteria: (1) <20% body mass loss with no recovery within 2 days; (2) inactivity, defined as no movement unless actively stimulated, on two consecutive days; (3) lack of grooming that worsened after 24 h; (4) enhanced Pause (Penh), a unitless index of lung function, of >2.5 times the animal's baseline; and/or (5) persistent hunched posture on two consecutive days.

### Observation frequency and schedule

Animals were followed for survival for up to 360 days after radiation exposure. Routine cage-side observations to assess gait, coat, behavior and activity were documented daily for the duration of the study. Animal body mass was assessed every 2 weeks throughout the study. Supportive care measures in the form of fluids, antibiotics and steroids were not provided in this study.

### Necropsy and tissue harvest

At the time of euthanasia, a bilateral thoracotomy was performed. The lungs and heart were removed, and pleural effusions measured as previously described. Lungs were separated (left versus right), and masses were individually collected and recorded. The left lung was rinsed in PBS, inflated with 10% neutral buffered formalin, and placed in 10% neutral buffered formalin for fixation. The three right lung lobes were separated and snap frozen in liquid nitrogen. Heart mass was collected and recorded. The heart was fixed in 10% neutral buffered formalin.

### Histopathology

Tissue sections (5 μm thick) were stained with Hematoxylin and Eosin (H&E) or Masson's trichrome at Charles River Pathology Associates (Frederick, MD). Scoring of fibrosis, alveolar and perivascular inflammation was performed by an independent observer blinded to animal strain, radiation dose and time of death. A board-certified pathologist at Charles River Pathology Associates, blinded to sample group, evaluated a subset of tissue sections to confirm findings.

### Animals and radiation exposure for differential gene expression analysis

Gene expression analysis with microarrays was performed as previously described (Jackson et al., 2016). Briefly, female C57BL/6J, CBA/J and C57L/J mice (Jackson Labs, Bar Harbor, ME) were irradiated at 10-12 weeks of age with 12.5 or 15 Gy of 320 kVp X-rays (Precision X-ray Inc.; HVL=2.00 mm Al, dose rate=67 cGy/m) at Duke University. Age-matched sham-irradiated mice were included as controls. Mice were euthanized 24 h post-exposure by pentobarbital overdose (>250 mg/kg). Lung tissue was excised, embedded in optimal cutting temperature (OCT) compound, and frozen over dry ice. Tissue was stored at −80°C until analysis.

### RNA isolation and affymetrix mouse gene chip hybridization

At the time of analysis, the right upper lobe from three to four mice per group was excised from OCT, placed in RNAlater (Fisher Scientific) for 5 min, and homogenized in 2 ml lysis buffer (QIAGEN, Valencia, CA) with zirconia-silica beads using a BeadBeater (BioSpec Products, Bartlesville, OK). RNA isolation was performed using the QIAGEN RNeasy kit according to the manufacturer's protocol with slight modifications (Barry et al., 2010). For gene expression analysis, samples were not pooled. Total RNA was assessed for quality with Agilent 2100 Bioanalyzer G2939A (Agilent Technologies, Santa Clara, CA) and Nanodrop 8000 spectrophotometer (Thermo Scientific–Nanodrop, Wilmington, DE). Hybridization targets were prepared with MessageAmp Premier RNA Amplification Kit (Applied Biosystems–Ambion, Austin, TX) from total RNA, hybridized to GeneChip Mouse Genome 430 2.0 arrays in Affymetrix GeneChip hybridization oven 645, washed in Affymetrix GeneChip Fluidics Station 450, and scanned with Affymetrix GeneChip Scanner 7G according to standard Affymetrix GeneChip Hybridization, Wash, and Stain protocols (Affymetrix, Santa Clara, CA).

### Data normalization and quality control for microarray analysis

To guard against batch effects or other technical factors impacting array data, we followed the following procedures. Animals were maintained under identical housing conditions and euthanized on the same day. Mice were irradiated in groups of 10 and alternated by strain along the radiation platform to minimize effects resulting from non-uniform radiation distribution or internal errors in the radiation procedure. Previous radiation field uniformity tests indicate <6% difference across the field. Samples were processed and hybridized in a single batch to protect against batch effects. RNA extraction and preprocessing methods used in this study are well characterized. To ensure reproducibility and minimize error, samples were not pooled but run independently. Gene expression values were normalized using robust multichip average (RMA) (Owzar et al., 2008). Unsupervised analysis, including principal component analysis (PCA) and hierarchical clustering, was performed to understand natural variations among the samples.

### Quantitative real-time PCR

Gene expression was validated using quantitative real-time reverse transcriptase PCR (ABI 7900HT, Applied Biosystems, Foster City, CA) as previously described (Xu et al., 2007). Briefly, the High Capacity cDNA Archive Kit (Applied Biosystems) was used according to the manufacturer's protocol to convert RNA to cDNA. Assays-on Demand Gene Expression primer sets were purchased from Applied Biosystems. Real-time PCR was performed using *Taq*Man Universal PCR Master Mix according to the *Taq*Man Gene Expression Assay protocol (Applied Biosystems). Relative gene expression was determined using the comparative CT method (ΔΔCT method). Data were analyzed using two-way analysis of variance (ANOVA) and multiple comparisons test.

### Western blot analysis of protein expression

The snap-frozen right lung lobe (*n*=5/group) was placed in a 2 ml tube filled with 1 ml zirconia-silica beads (BioSpec Products) and 2 ml ice-cold homogenization buffer (1% sodium deoxycholate, 5 mM Tris-HCl pH 7.4, 2 mM EDTA, 10 mg/ml aprotinin, 0.5 mM phenylmethylsulfonyl fluoride, 1 mM pepstatin A, 0.1 mg/ml benzamidine with or without phosphatase inhibitors). Tissue was then homogenized using the Mini-Beadbeater (BioSpec Products). Protein concentration was determined using the Nanodrop Spectrophotometer (Thermo Scientific). Western blot was performed as previously described (Zhang et al., 2012). The anti-alpha-1 antitrypsin antibody was purchased from Abcam, Cambridge, MA (AB43105; 1:1000) (Chambers and Johnston, 2003), and for alpha-1 acid glycoprotein was purchased from R&D Systems, Minneapolis, MN (MAB5934; 1:2 mg/ml). To control for loading efficiency, blots were stripped and reprobed with GAPDH or α-tubulin antibody (Sigma-Aldrich, Billerica, MA). Differences between groups were analyzed by Student’s *t*-test.

### Transmission electron microscopy

C57L/J and C57BL/6J mice were irradiated to the whole thorax with a single dose of either 0 Gy or 15 Gy using the dosimetric parameters described above. Twenty-four hours later, animals were euthanized by sodium pentobarbital overdose (>250 mg/kg), and a bilateral thoracotomy was performed. Lung tissue was extracted, the lobes separated, and the left lobe fixed with 10% neutral buffered formalin. The left lung was then cut into longitudinal sections for electron microscopy or paraffin embedding. Paraffin embedded tissue was cut into 5 μm-thick sections and stained with H&E for evaluation of histopathological damage as described above. For transmission electron microscopy, the Duke Electron Microscopy facility, a shared resource, prepared the tissue as previously described (Miller et al., 1976). Briefly, thick sections were cut (0.5 μm) and stained with Toluidine Blue. Sections were visualized under a light microscope to determine the location for ultrathin sections. Mild inflammation was seen in C57L/J thick sections but not in C57BL/6J sections. Ultrathin sections were stained with uranyl acetate and lead citrate and visualized using a Philips CM12 transmission electron microscope.

### Statistical analysis for natural history of disease progression

Statistical analysis was performed at the Indiana University School of Medicine (Indianapolis, IN) using SAS version 9.3. Proportions of mice that survived to day 360 were presented by radiation dose groups and by radiation dose and sex. A logistic regression model for 360-day mortality was fit to examine the association of radiation dose and mortality by day 360 adjusted for sex. The model included radiation dose, sex, and the radiation dose by sex interaction effect in the model. Radiation dose was included in the model as a continuous variable. Lethal doses (i.e. LD10/360-LD99/360) and their 95% confidence intervals were estimated using logistic regression models for 360 day mortality with the natural log of radiation dose. A dose-response curve was plotted. A Cox proportional hazard regression model was fitted to compare time to death between radiation dose groups adjusted for sex. The radiation dose by sex interaction effect was also tested. Kaplan–Meier survival curves were presented by radiation dose groups. Median survival times were estimated by radiation dose group. All statistical analyses were conducted as two-sided tests at 0.05 significance levels. One-way ANOVA was utilized to compare differences in gene expression among strains, radiation doses, and response (acute pneumonitis versus delayed injury) types, separately. Two-way ANOVA was used to compare the strain and radiation dose effect simultaneously.
